# Transition between individually different and common features in skilled drumming movements

**DOI:** 10.3389/fspor.2022.923180

**Published:** 2022-07-26

**Authors:** Ken Takiyama, Masaya Hirashima, Shinya Fujii

**Affiliations:** ^1^Department of Electrical Engineering and Computer Science, Tokyo University of Agriculture and Technology, Tokyo, Japan; ^2^Center for Information and Neural Networks (CiNet), National Institute of Information and Communications Technology, Osaka University, Osaka, Japan; ^3^Faculty of Environment and Information Studies, Keio University, Fujisawa, Japan

**Keywords:** tensor decomposition, individual differences, movement phase, skilled movements, drumming movements

## Abstract

Why do professional athletes and musicians exhibit individually different motion patterns? For example, baseball pitchers generate various pitching forms, e.g., variable wind-up, cocking, and follow-through forms. However, they commonly rotate their wrists and fingers at increasingly high speeds *via* shoulder and trunk motions. Despite the universality of common and individually different motion patterns in skilled movements, the abovementioned question remains unanswered. Here, we focus on a motion required to hit a snare drum, including the indirect phase of task achievement (i.e., the early movement and mid-flight phases) and the direct phase of task achievement (i.e., the hit phase). We apply tensor decomposition to collected kinematic data for the drum-hitting motion, enabling us to decompose high-dimensional and time-varying motion data into individually different and common movement patterns. As a result, individually different motion patterns emerge during the indirect phase of task achievement, and common motion patterns are evident in the direct phase of task achievement. Athletes and musicians are thus possibly allowed to perform individually different motion patterns during the indirect phase of task achievement. Additionally, they are required to exhibit common patterns during the direct phase of task achievement.

## Introduction

Skilled movements entail two types of features: features that are invariant across individuals and features that differ across individuals. For example, in baseball pitching, baseball players commonly rotate their distal wrists and elbows at increasingly high speeds *via* proximal motions in the trunk and shoulders (Hirashima et al., 2008). In walking, the trajectory of the center of mass (CoM) shows common speed-dependence across subjects (Orendurff et al., 2004; Takiyama et al., 2020a); specifically, the width in the right-left direction decreases while the height in the up-down direction increases as walking speed increases. These modulations of CoM trajectories possibly enable us to walk efficiently (Cavagna et al., 1963; Cavagna and Kaneko, 1977). In switching between walking and running, peak timing of muscle activities show dependence on locomotion mode (i.e., either walking or running; Cappellini et al., 2006; Takiyama et al., 2020b), which enables leg muscles to be activated at appropriate timings for stable and efficient locomotion. In roll and rise tasks, common motion features across subjects emerge depending on task constraints (Kuniyoshi et al., 2004). The study also demonstrated that robots could perform the same roll and rise task while utilizing common motion features. Thus, common motion features play essential roles in achieving several motion patterns.

In addition to common motion features, we can confirm individual differences in most motion patterns. For example, every pitcher throws a ball by moving through various wind-up, cocking, and follow-through motions. The unique motion patterns across subjects originated from numerous numbers of degrees of freedom (DoFs) inherent in our body. In several situations, we have more DoFs than necessary to achieve planned movements (Bernstein, 1967). Due to the significant number of DoFs, the same action is achievable *via* a variety of movement patterns rather than a unique movement pattern, and this concept is referred to as redundancy (Bernstein, 1967). Let us assume a case in which all subjects grasp a drumstick by their right hands with the same relative posture to their wrists. When they hit a snare drum by moving only their wrist, their motions are close to each other. In other words, one DoF (i.e., a wrist joint angle) is sufficient to achieve this task. If they are allowed to move their elbow (i.e., two DoFs), they have multiple movement choices: move both the elbow and wrist, only the elbow, or only the wrist. Individually different motion patterns thus originate from more DoFs than necessary to achieve planned movements.

The individual different motion features are observable along with common motion features in multiple movement patterns. In playing the piano, pianists modulate kinematic parameters depending on keystroke loudness in different manners (Furuya et al., 2012). However, they commonly exhibit speed-invariant kinematics (Furuya and Soechting, 2012). In baseball pitching, pitchers modulate their muscle activities to throw the ball with 100% motion effort in different ways from each other (Hashimoto et al., 2021). However, when pitchers increased their motion effort from 50 to 80%, the way of modulating their muscle activities is common among them (Hashimoto et al., 2021). In hitting a snare drum repetitively with a constant time interval, percussion players hit the drum with individually different motion trajectories (Dahl, 2004). However, they demonstrate common motion features, namely, preparatory height determines hitting velocity (Dahl, 2004).

Taken together, common and individually different motion features coexist. The coexistence is evident in several motion patterns, such as in baseball pitching (Hashimoto et al., 2021), roll-and-rise motions (Kuniyoshi et al., 2004), drum hitting (Fujii et al., 2009, 2010), running (Phinyomark et al., 2015), and piano playing (Furuya et al., 2012).

Despite the commonality of invariant and individually different features among several motion patterns, how they are interrelated in skilled movements remains unclear. A challenge in investigating the common and individually different patterns is determining how to extract these features from high-dimensional and time-varying motion data. A possible solution is to quantify these patterns on the basis of low-dimensional structures or modules inherent in time-varying motions (Bernstein, 1967; Bizzi et al., 1991; Borghese et al., 1996; Cappellini et al., 2006; Takiyama et al., 2020b; Hashimoto et al., 2021). A working hypothesis is that human motor systems control joint kinematics and muscle activities synergistically rather than independently. Because the inherent number of DoFs in our body is tremendous, a method to reduce the DoFs is to group joints and muscles. Groups of joints or muscles are referred to as spatial modules, which have been reported to exist in leg movements of frogs (Bizzi et al., 1991; d'Avella et al., 2003), human locomotion (Borghese et al., 1996; Ivanenko et al., 2004), standing (Torres-Oviedo and Ting, 2007), arm-reaching movements (d'Avella et al., 2006), piano-playing motions (Furuya et al., 2011), and cycling (Barroso et al., 2014). Time-varying recruitment patterns of spatial modules are referred to as temporal modules. By extracting these modules and reducing the dimensions in motion data, we discuss how these modules are common or individually different across drummers.

**Figure 1:**
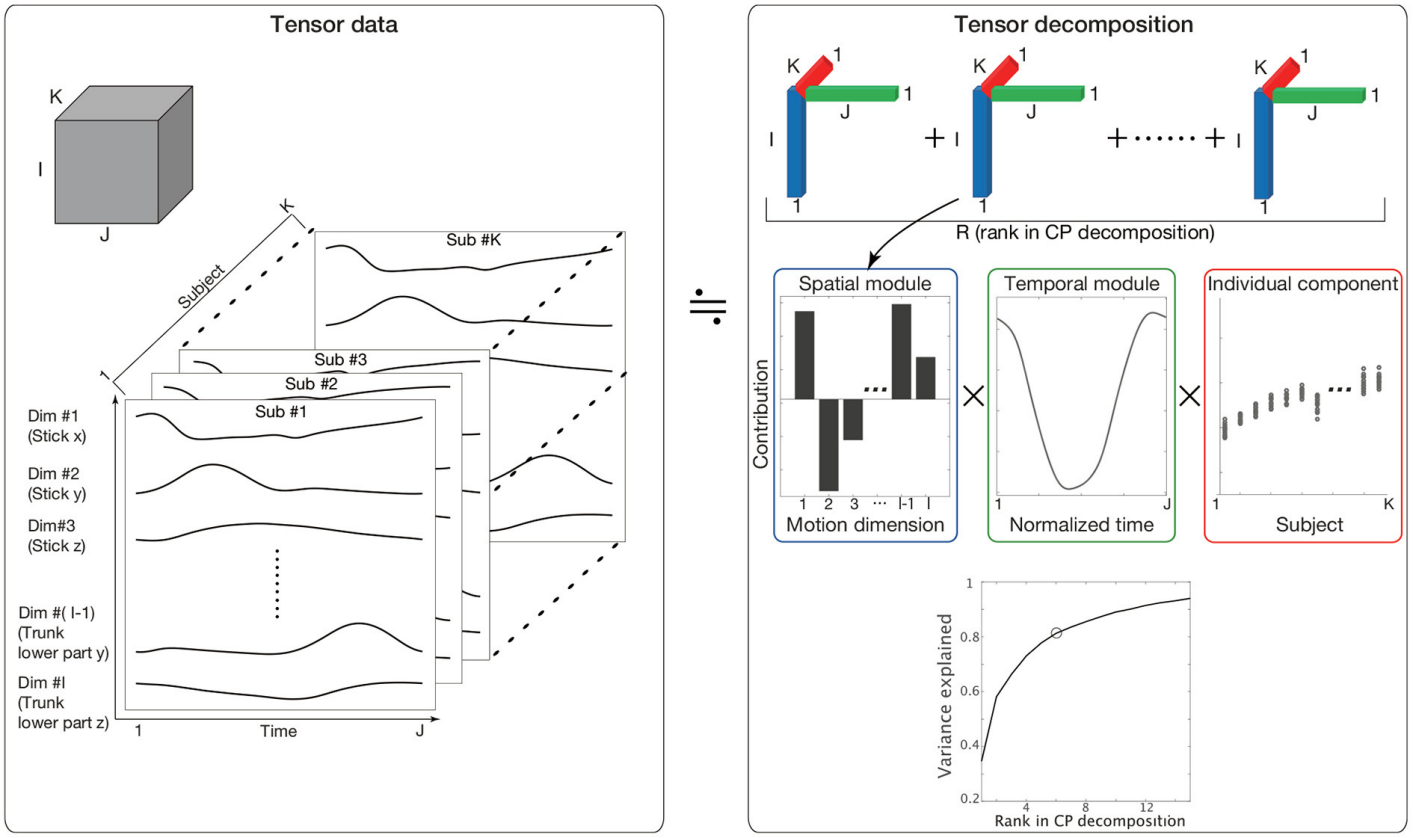
The concept of CP decomposition. The three-dimensional array data consisting of *S* columns, *T* raws, and *K* slices are decomposed into combinations of spatial modules, which are denoted by bar graphs in the blue frame; temporal modules, which are denoted by line plots in the green frame; and individual components, which are denoted by circle dots in the red frame. (Lower-right) The variance in the original data explained by CP decompositions. The vertical axis indicates the proportion of the original variance explained by CP decomposition. The horizontal axis indicates the number of tensors used in CP decomposition. Because we focused on 80% criteria, we selected six tensors in the current study. Of note, some important results were invariant independent of the criteria, as discussed later.

To quantify similarities and differences in spatiotemporal modules among individuals, tensor decomposition (Kolda and Bader, 2009) is an effective method. Methods commonly used to evaluate spatiotemporal modules include matrix decomposition methods, such as principal component analysis (PCA) and non-negative matrix factorization (NNMF) (Borghese et al., 1996; Lee and Seung, 1999; Ivanenko et al., 2004; Torres-Oviedo and Ting, 2007). These methods enable us to evaluate two features (e.g., spatial and temporal modules) by analyzing matrices with two elements (i.e., rows and columns). In other words, these methods are not always suitable for analyzing more than two factors at once. In the current study, we quantified three factors: spatial modules, temporal modules, and the similarities of these modules across individuals. For the analysis of (more than) two factors, tensor decomposition rather than matrix decomposition is suitable (Kolda and Bader, 2009). Three-dimensional tensor decomposition consists of slices of matrices along a third dimension (the dimension along K in the left panel in Figure 1). In setting columns (I), rows (J), and the third dimension (K) to include spatial, temporal, and individual information, respectively, Candecomp/Parafac (CP) decomposition enables us to extract spatial and temporal modules and determine how each spatiotemporal module is recruited in each individual (the upper-right and middle-right panels in Figure 1).

The third set of features extracted *via* CP decomposition (i.e., a feature to indicate how each spatiotemporal module is recruited in each individual) is hereafter referred to as individual components. If all subjects recruited a pair of spatial and temporal modules in the same way, the associated individual components could have the same value in all subjects, i.e., a small variability. If all subjects recruited a pair of spatial and temporal modules in a completely different manner, the associated individual components could have different values in all subjects, i.e., a larger variability. The variabilities of individual components thus enable us to assess whether the associated spatial and temporal modules are common or individually different motion components.

Here, we investigated relationships between common and individually different motion patterns from the perspective of the low-dimensional structure inherent in motion data. To reveal the relationship, we focused on the kinematic data associated with professional drummers hitting a snare drum. This motion includes (at least) three phases: (1) the early movement phase [i.e., raise a drumstick to set the preparatory height], (2) mid-flight phase [i.e., swing the stick to hit the snare drum], and (3) drum-hitting phase [i.e., hit the snare drum]. When the primary task is to hit a snare drum, the early movement phase and mid-flight phase are indirectly related to the task achievement, and the hit phase is directly related to the task achievement. The drum-hitting motion thus enables us to discuss how common and individually different motion patterns emerge in both the direct and indirect phases of task achievement.

In summary, by analyzing drum-hitting motions, we expect to determine how common and individually different patterns are interrelated depending on the motion phase. By utilizing CP decomposition, the current study reveals the phase-dependence of common and individual motion features.

## Materials and methods

### Participants

Seventeen professional drummers (15 males and two females) participated in the experiment. All drummers were right-handed; the handedness score, assessed using the Edinburgh Handedness Inventory, was 79.4 ± 21.9 (mean ± standard deviation) and ranged from 40 to 100. The mean age was 40.1 ± 8.1 years (range = 23–58). Participants began drum training at 13.9 ± 3.0 years of age (range = 6–17) and had 26.2 ± 9.4 years of experience (range = 8–43). Supplementary Table 1 shows more detailed attributes of each subject. All of the drummers had released numerous CDs and performed in many live concerts as members of professional bands or as support musicians for professional singers. In accordance with the Declaration of Helsinki, the participants received clear explanations of the experimental procedure and provided written informed consent prior to participating in the study. The experimental procedure was approved by the Ethical Committee of the Graduate School of Arts and Sciences of the University of Tokyo.

### Apparatus and preprocessing

A snare drum (14-inch diameter, Carbon-Ply-Maple Series, Pearl) was located in front of the participants. The height and position of the snare drum were adjusted to fit each participant, enabling him or her to hit the drum comfortably with his or her right hand while holding a drumstick. Sixteen drummers used a drumstick (190STH Standard Hickory, Pearl) prepared by the experimenter, while one drummer used his own stick (118 M Maple, Pearl) since he felt uncomfortable using the stick prepared by the experimenter. The time at which the drum was hit was detected from the S3 marker on the stick frame by frame on the Eva RealTime software (v4.7.20, Motion Analysis Corporation, Santa Rosa, CA, USA). We detected the frame at which the y-coordinate (up-down) data of the S3 marker changed the direction from downward to upward and the detected frame was the last time point in the plot.

**Figure 2:**
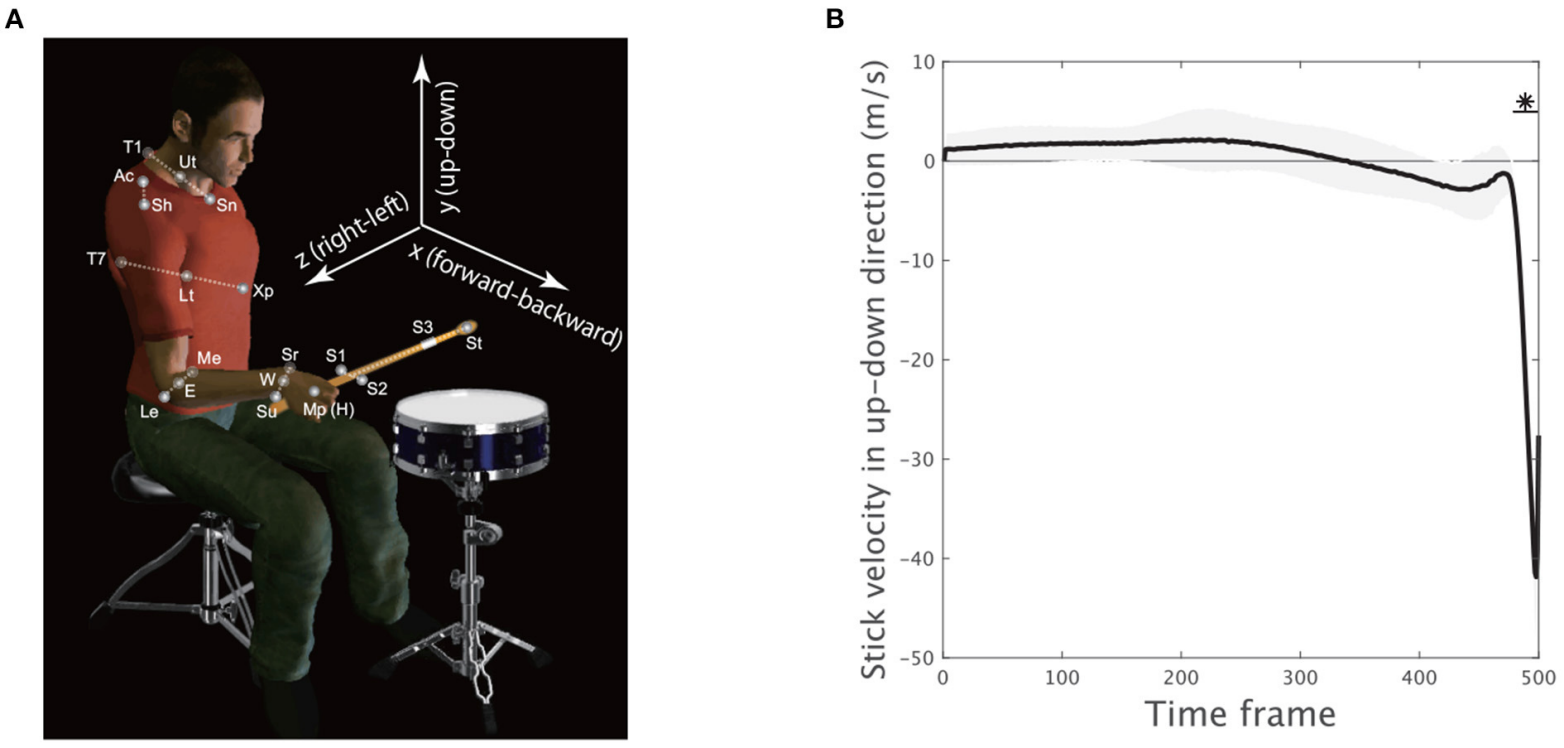
Experimental setup and movement variability. **(A)** Four markers were located at the sternal notch (Sn), T1 spinal process (T1), xiphoid process (Xp), and T7 spinal process (T7) to track trunk movement. Six markers were located at the acromion (Ac), lateral epicondyle (Le), medial epicondyle (Me), styloid process of the ulna (Su), styloid process of the radius (Sr), and middle metacarpophalangeal joint (Mp) to track upper limb motion. Three markers were located on the drumstick to capture stick motion (S1, S2, and S3). The midpoint Ut between Sn and T1, the midpoint Lt between Xp and T7, the midpoint E between Me and Le, and the midpoint W between Sr and Su were calculated. The center of the shoulder (Sh) joint was estimated from the acromion (Ac) marker. The Mp marker was used to detect the position of the hand (H). The position of the tip of the stick (St) was calculated using markers S1–S3. **(B)** The stick velocity in the up-down direction. The solid black line indicates the velocity averaged across all the trials and the subjects. The black shaded area denotes the standard error of the mean of the velocity averaged across all subjects. The horizontal solid black lines with asterisks indicate a significant difference in the velocity from 0 in the marked time frames (*p* < 0.01, paired *t*-test with Bonferroni's correction).

Spherical reflective markers were used to track the motion of the upper body (Figure 2A). Four markers were located at the sternal notch (abbreviated as Sn), T1 spinal process (T1), xiphoid process (Xp), and T7 spinal process (T7) to capture the movement of the trunk. Six markers were located at the acromion (Ac), lateral epicondyle (Le), medial epicondyle (Me), styloid process of the ulna (Su), styloid process of the radius (Sr), and middle metacarpophalangeal joint (Mp) to capture the motion of the upper limb. Three markers were located on the drumstick to capture the motion of the stick (S1, S2, and S3). Marker position data were recorded at 200 Hz by an optical motion capture system (Motion Analysis Corporation, Santa Rosa, CA, USA). The position data of the markers on the stick were not smoothed to preserve the magnitude of impact reflected in the data. The other marker data were smoothed by applying a bidirectional, fourth-order, low-pass Butterworth filter. The cutoff frequency was calculated for each marker by residual analysis (Winter, 1990). To reduce numerical differentiation error, the data were resampled at 1,000 Hz by spline interpolation. We calculated the midpoint Ut between Sn and T1, the midpoint Lt between Xp and T7, the midpoint E between Me and Le, and the midpoint W between Sr and Su (Figure 2A). The shoulder marker Ac was incorporated to calculate the center of the shoulder joint (Sh) according to the methods in a previous study (Fleisig et al., 1996). The Mp marker was used to track the position of the hand (H). The tip of the stick (StickEnd) was calculated using the positions of markers S1–S3 attached to the stick.

### Procedure

We instructed the participants to hit a snare drum once in a trial while gradually increasing their movement effort from their minimal to maximal levels in each trial (see Supplementary Figure 3). Subjects determined the number of trials in each set and the duration of each movement in each trial by themselves. A total of five sets of trials were performed. We did not explicitly restrict the number of trials in each set. Each participant decided by themselves how many trials they performed within each set. We analyzed the drum-hitting motions with the minimum effort, i.e., in the first trial in each set, and the motions with the maximum effort, i.e., in the final trial in each set, while averaging these motions across the five sets. Because the number of trials was different for each drummer, the effort exerted for each trial was non-uniform, except for in the first and final trials. We thus focused on drum-hitting motions performed with minimum or maximum effort by each subject.

### Tensor decomposition

Tensor decomposition enables us to assess more than two factors through the analysis of multidimensional array data. In the current study, a major aim was to assess spatial modules (i.e., a set of spatial positions of body parts), temporal modules (i.e., spatial modules' recruitment time course), and how these modules were recruited in each subject. To assess the three factors at once, we utilized tensor decomposition.

Kinematic data were preprocessed as tensor data *X*_*i, j, k*_, where *i* indicated the *i*th motion dimension [e.g., *i* = 1, …, *I*, the position of the *l*th marker in the *x*-coordinate plane is the (3 × *l*−2)th dimension, that in the y-coordinate plane is the (3 × *l*−1)th dimension and that in the z-coordinate plane is the (3 × *l*)th dimension (Figure 1)], *j* indicated the *j*th time frame (*j* = 1, …, *J*), and *k* indicated the *k*th participant (*k* = 1.., *K*). The kinematic data for each subject were standardized such that the mean and variance of each motion dimension equaled 0 and 1, respectively, across all the time frames. This standardization procedure enabled us to assess each motion dimension fairly. Without the standardization, the across-trial variabilities were mainly based on the body parts with large movements (e.g., stick, wrist, or elbow) and the subjects with a long arm length and large movements, which introduces bias into our discussion of spatial modules, temporal modules, and individual components among subjects and anatomical landmarks. Of note, this standardization process did not affect our results because it is possible to reconstruct the original marker position data by addition and multiplication processes (see Supplementary Material and Furuki and Takiyama, 2019).

*X*_*i, j, k*_ was decomposed as

$X_{i,j,k}≃\sum_{r\;=\;1}^{R}\;\lambda_{r}s_{i,r}t_{j,r}u_{k,r},$

where **s**_*r*_ = (*s*_1, *r*_, *s*_2, *r*_, …, *s*_*I, r*_) indicated the *r*th spatial module, **t**_*r*_ = (*t*_1, *r*_, *t*_2, *r*_, …, *t*_*J, r*_) indicated the *r*th temporal module, and **u**_*r*_ = (*u*_1, *r*_, *u*_2, *r*_, …, *u*_*K, r*_) indicated how the pair of the *r*th spatiotemporal module was recruited in each subject (Figure 1). Throughout this study, we refer to **u**_*r*_ as an individual component. Additionally, the current study refers to a combination of the spatial module, temporal module, and individual component as a tensor. Under the normalizations |**s**_*r*_| = 1, |**t**_**r**_| = 1, and |**u**_*r*_| = 1, the scaling factor λ_*r*_≥0 indicated the contribution of the *r*th tensor to explain the original data, and *R* indicated the number of decomposed tensors. The scaling factor λ_*r*_ was defined as a positive value. The spatial modules, temporal modules, and individual components were estimated while minimizing the squared error $E=\frac{1}{2IJK}\underset{i,j,k}{\sum }{\left(X_{i,j,k}-\overset{R}{\underset{r=1}{\sum }}\lambda_{r}s_{i,r}t_{j,r}u_{k,r}\right)}^{2}$. Throughout this study, we utilized MATLAB 2019a (MathWorks, Natick, Massachusetts) and the function “cp_als” (alternating least square; Kolda and Bader, 2009) *via* the tensor toolbox in MATLAB (Bader and Kolda, 2017).

The kinematic data of the *k*th subject were decomposed as


$$X_{:,:,k}≃\sum {}_{r=\;1}^{R}\lambda_{r}s_{r}^{T}t_{r}u_{k,r}.$$

This equation indicated that the spatial and temporal modules were independent of *k* and common across all the subjects. The individual component *u*_*k, r*_ determined how these modules were recruited by each subject. Thus, assessing individual differences inherent in the kinematic data based on the individual components was natural.

We selected the number of tensors *R* based on the criteria to explain more than 80% of the variance in the original data. As discussed in the Results Section, our results are invariant independent of the criteria. Equivalently, we selected *R* to be the minimum number required to exceed a value of 0.8 for the uncentered coefficient of determination (Hashimoto et al., 2021). Although 80% accuracy likely seems to be a lower accuracy criterion than those used in previous studies using PCA or NNMF, the lower criterion of CP decomposition is dependent on a small number of parameters. In PCA, for example, when common spatial modules are extracted across all the subjects, a matrix whose size is *I*×(*J*×*K*) must be analyzed. The *R* principal components include *R*×*I*+*R*×(*J*×*K*) parameters. In our case, *I* = 21, *J* = 500, *K* = 17, and *R* = 6. With these parameters, although orthogonal constraints can decrease the number of parameters to a certain extent, PCA yields ~51,126 parameters. When applying PCA to each subject separately, the number of parameters is (*R*×*I*+*R*×*J*) × *K* = 53, 142. In contrast, CP decomposition requires *R*×(*I*+*J*+*K*) = 3, 228 parameters. These significant differences in the number of parameters can lead to a lower percent of variance being explained in CP decomposition than in PCA with the same number of modules.

Of note, there is no task or biomechanical constraint on CP decomposition itself. However, CP decomposition yields spatial modules, temporal modules, and individual components to approximate the original motion data, including task constraint and biomechanical constraint. Motion data include the biomechanical constraint because this is data related to human motion (in our case) following the biomechanical constraint. In addition, we frequently measured motion data when subjects performed some tasks; therefore, motion data include the task constraint. Extracted spatial modules, temporal modules, and individual components thus possibly and indirectly include biomechanical constraints.

### Clustering

The current study used a hierarchical clustering method for individual components **u**. Because six tensors were extracted, the input **u** was a six-dimensional value for each subject. We relied on the MATLAB function “evalcluster” with tree-type hierarchical clustering, gap value, Euclidean distance, and globalMaxSE criteria to select the number of clusters.

## Results

To investigate how spatiotemporal modules are common or individually different depending on movement phases (i.e., either in the early movement, mid-flight, or hit phase), we applied CP decomposition (Kolda and Bader, 2009) to professional drum-hitting motions performed with maximum effort [*N* = 17 (Figures 1, 2A)]. The current study focused on motions performed with maximum effort because this subjective level of effort can be uniform in our setting (see Section Materials and methods for details).

The current study analyzed seven anatomical landmarks (StickEnd [abbreviated as S], Hand [H], Wrist [W], Elbow [E], Shoulder [Sh], Upper Trunk [Ut], and Lower Trunk [Lt]) while focusing on motions in three-dimensional space in the x- (forward-backward), y- (up-down), and z-coordinates (right-left; Figure 2A, detailed descriptions of the data collection are provided in the Materials and methods Section). Of note, the following results were invariant when we analyzed joint angles (see Supplementary Material). We analyzed the positions of the seven anatomical landmarks over 1,000 time frames (i.e., 1 s) before the stick hit the snare drum (details on the detection of hit timings are provided in the Materials and methods Section). To reduce the computational time, we analyzed every other frame (i.e., the number of analyzed frames was 500, and the 500th time frame corresponded to the time of impact).

### Estimation of the direct and indirect phases of task achievement through evaluating the downward velocity of the drum-stick required to hit a snare drum

To confirm how the early movement, mid-flight, and hit phases were related to drum-hitting motion, we calculated the vertical velocity of the drumstick (S) because maximizing it downward at the drum surface was a task requirement. Because each subject performed drum-hitting motions five times, the current study averaged the velocity profiles across the trials in each subject. We then calculated the mean of the velocity profile across the subjects (the solid black line in Figure 2B). The asterisk with the horizontal solid black line shows the time frame when the up-down stick velocity among the subjects was significantly different from 0 (*p* < 0.01 [corrected]), indicating that the direct phase of task achievement was observable only during the very final phase (i.e., the 480th−500th time frames).

Due to the stick up-down velocity, we regarded both the early movement and mid-flight phase as indirect phases (i.e., the 1st−479th time frames) and the movement termination as a direct phase of task achievement (i.e., 480th−500th time frames). Let us hereafter refer to both the early movement and mid-flight phases as the indirect phase and refer to the drum-hitting phase as the direct phase. The current study examined how skilled motions incorporate common and individually different motion features with respect to the direct phase and indirect phase to perform tasks.

### Assessment of common and individual motion components *via* CP decomposition

CP decomposition yielded six combinations of spatial modules, temporal modules, and individual components (i.e., how the modules are recruited in each subject) with certain criteria to explain more than 80% of the variance in the original data (the lower right panel in Figure 1; the dependence on the criteria is mentioned in a later section). An advantage of this method is that motion data can be decomposed into spatial, temporal, and individual information, which enables us to understand how each individual moves each group of body parts in each motion phase from high-dimensional, time-varying, and multiple-subject motion data.

**Figure 3:**
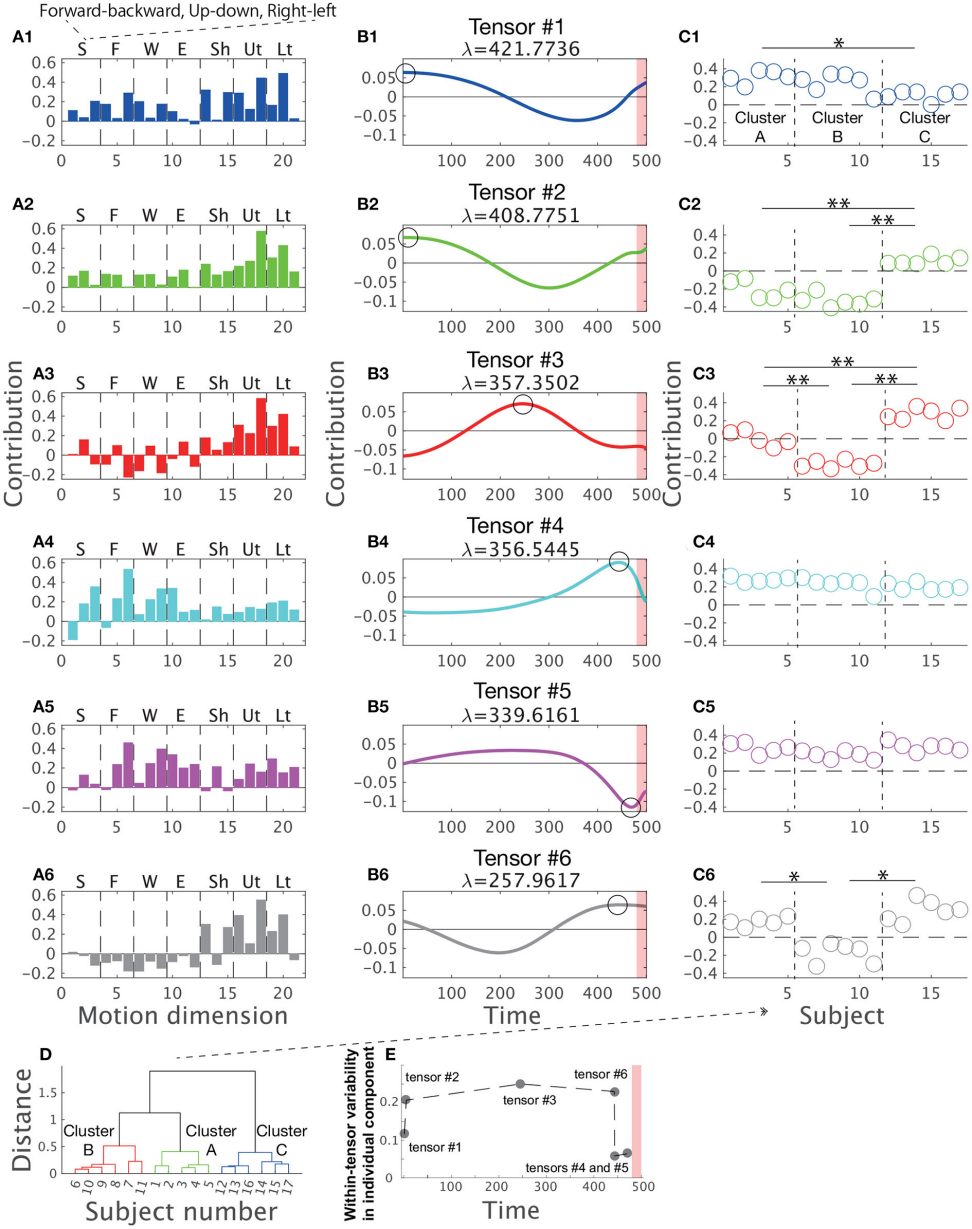
Tensors extracted *via* CP decomposition and clustering of individual components. The current study applied CP decomposition to the motion data to hit a snare drum with maximal effort. The bar graphs indicate spatial modules **(A1–A6)**, the line plots denote temporal modules **(B1–B6)**, and the scatter plots show individual components **(C1–C6)**. λ is a scaling factor indicating how each tensor contributes to reconstructing original data. All the spatial modules, temporal modules, and individual components were normalized such that each norm equals 1. (Left) In the spatial modules, S represents stick, F represents finger, W represents wrist, E represents elbow, Sh represents shoulder, Ut represents the upper part of the trunk, and Lt represents the lower part of the trunk. In each segmentation, three bins indicate the marker positions in the x- (forward-backward direction), y- (upward-downward direction), and z-coordinates (rightward-leftward direction) from left to right. (Middle) In the temporal modules, the 500th time frame corresponds to the time at which the drum was hit. Each black circle indicates the time of the peak of each temporal module (i.e., the maximum absolute value). Non-standardized motions at these peak timings are shown in Figure 5. The red shaded areas in the same panels demonstrate the phases when the magnitude of the stick up-down velocity was not equal to 0 (see Figure 2B). **(C1–C6)** The individual components indicate how spatiotemporal modules are recruited by each subject. Of note, subject number was sorted based on the cluster number in a *post-hoc* manner to increase visibility. Each cluster is separated by the horizontal dotted black lines. The single and double asterisks above the horizontal solid black lines indicate significant differences *via* Tukey's comparison test at *p* < 0.05 and *p* < 0.01, respectively. **(D)** The associated subject number in each cluster. **(E)** Within-tensor variability of each tensor based on the peak timing of each temporal module.

Figure 3 shows the combinations extracted by CP decomposition. For example, the blue bar graph A1 indicates a set of spatial positions of body parts (i.e., spatial module), the blue line plot B1 indicates its recruitment time course (i.e., temporal module), and the scatter plot C1 indicates the degree of how the spatiotemporal module is recruited for each subject (i.e., individual components). A set of combinations of the three components is referred to as tensor hereafter. The order of the tensors depended on the contribution to explaining the original data, denoted by λ (see the Materials and methods Section for details). An important feature of CP decomposition is that modules or components in a certain tensor are related only within each tensor. The colors in Figure 3 show these relations within each tensor: the blue-colored spatial module is related to the blue-colored temporal module and individual components, and vice versa, but it is not related to the green-, red-, or cyan-colored spatial modules, temporal modules, or individual components. Throughout this study, we denoted spatial modules as bar graphs, temporal modules as line plots, and individual components as scatter plots in accordance with the style used in previous studies (Williams et al., 2018; Takiyama et al., 2020b; Hashimoto et al., 2021). The circles in the panels of the temporal modules (Figures 3B1–B6) indicate the peak timings (i.e., the maximum absolute values). The red shaded areas in the same panels demonstrate the phases when the magnitude of the stick up-down velocity was different from 0, i.e., the direct phase of task achievement (Figure 2B). The vertical dotted black lines in Figures 3C1–C6 separate the different clusters mentioned later. Of note, CP decomposition allows similarity of spatial modules, temporal modules, and individual components among tensors because orthogonal constraint is not necessary in contrast to PCA.

Tensor #1 is denoted in blue in Figure 3. The spatial module mainly involved up-and-down motions of the lower region of the trunk and right-and-left motions of the upper region of the trunk (Figure 3A1). The temporal module indicated the recruitment of the spatial module mainly in the indirect phase (Figure 3B1). The peak timing of the temporal module indicated larger recruitment of the spatial module in the early part of the indirect phase. The individual component indicated recruitment of the associated spatiotemporal module mainly in subjects #1 to #10 but not in subjects #11 to #17 (Figure 3C1).

Because CP decomposition does not require orthogonal constraints, the spatial modules in tensors #2 (green color), #3 (red color), and #6 (black color) are similar to the spatial module in tensor #1, especially in the recruitment patterns of the trunk in the spatial modules (Figures 3A2,A3,A6; the absolute value of Pearson's correlation coefficient was 0.7395 ±0.1910 [mean ± standard deviation, *p* < 0.0420]). Of note, CP decomposition results in unique solutions, except when different signs are considered. Because CP decomposition provides six sets of a spatial module times a temporal module times an individual component, the decomposition process provides the same solutions as multiplying both the spatial and temporal modules by −1, multiplying both the spatial modules and individual components by −1, or multiplying both temporal modules and individual components by −1. We must therefore interpret each tensor while considering the signs.

Despite the high correlations in the spatial modules among tensors #1, #2, #3, and #6, the temporal modules in these tensors have different characteristics (Figures 3B1–B3,B6). In particular, the peak timings differ from each other, i.e., in tensor #1 and #2, the spatial module was recruited at the early part of the indirect phase (Figure 3B2) and both tensor #3 and #6 showed the largest activities at the middle part of the indirect phase (Figures 3B3,B6). Although tensors #1 and #2 have similar temporal modules, the timing of the peak was faster in tensor #1 (the first time frame) than in tensor #2 (the fifth time frame) and the second peak was faster in tensor #2 (301st time frame) than in tensor #1 (357th time frame). Furthermore, the spatial modules in tensors #1 and #2 were different, i.e., the temporal modules in tensors #1 and #2 were associated with different groups of body and stick parts. In summary, the spatial modules in tensors #1, #2, #3, and #6 showed sequential recruitment patterns within the indirect phase from tensors #1 and #2 to tensors #3 and #6.

Among these tensors, we also found differences in the individual components, especially in tensors #2, #3, and #6 (Figures 3C2,C3,C6). For example, in tensor #3, subjects #12–#17 recruited associated spatiotemporal modules to a large extent, but subjects #1 to #5 recruited these modules to a small extent (Figure 3C3). These results indicated that tensors #2, #3, and #6 corresponded to individually different motion features related to early movement and mid-flight.

In contrast, the spatiotemporal modules in tensors #4 (cyan color) and #5 (magenta color) were commonly recruited across all the subjects (Figures 3C4,C5). The right-and-left motions of the finger, the right-and-left motions of the wrist, and the forward-and-backward motions of the elbow were evident in these spatial modules. In tensor #4, the right-left motion of the tip of the stick was also obvious. The peak timings of temporal modules demonstrated the maximal recruitment of the spatial modules near the direct phase indicated by the red shaded areas. In contrast to tensors #1, #2, #3, and #6, there were few differences in the individual components (Figures 3C4,C5). These results denoted that the spatiotemporal modules in tensors #4 and #5 are motion features that are common across professional drummers.

In summary, we confirmed individual differences near the early and middle parts of the indirect phase based on tensors #1, #2, #3, and #6. In contrast, few individual differences existed near the direct phase based on tensors #4 and #5.

### Phase-dependent transition between individually different and common features

To assess the individual differences in more detail, we applied a hierarchical clustering method to the individual components (see section Materials and methods for details). The number of clusters can be used as a measure of individual differences. If every subject showed completely different movements, the number of clusters would equal the number of subjects. If each subject showed the same exact movements, the number of clusters would be one. An intermediate number of clusters indicated that professional drum-hitting motions with maximum effort were classified into groups.

The number of clusters inherent in the individual components was three based on gap value criteria (Supplementary Figure 2A). Figure 3D shows the divided clusters and associated subject numbers. To gain insight into how the clusters were divided based on tensor information, we performed two-way (cluster × tensor) ANOVA on individual components, which revealed not only a significant main effect of cluster number [*F*_(2, 84)_ = 99.27, *p* = 7.48 ×10^−23^] and tensor number [*F*_(5, 84)_ = 78.75, *p* = 3.17 × 10^−30^] but also an interaction between cluster number and tensor number [*F*_(10, 84)_ = 27.83, *p* = 1.27 × 10^−22^]. These results indicated that the individual components differed among clusters, the individual components differed among tensors, and the difference in individual components among clusters depends on tensor number. To examine the difference in individual components among clusters in each tensor, we performed Tukey's *post-hoc* multiple comparison test for the individual components in each tensor. In the order of peak timings in each temporal module, at the first peak (tensor #1), a difference was found between clusters A and C (Figure 3C1, *p* = 0.0123). At the second peak (tensor #2), the differences in clusters A and B from cluster C were obvious (Figure 3C2, *p* = 7.24 × 10^−7^ and *p* = 7.05 × 10^−7^). At the third peak (tensor #3), the differences were evident among the three clusters (*p* = 1.24 × 10^−6^ [between clusters A and B], *p* = 2.09 × 10^−6^ [between clusters A and C], and *p* = 7.00 × 10^−7^ [between clusters B and C]). At the fourth peak (tensor #6), differences in clusters A and C from cluster B were identified (Figure 3C6, *p* = 7.01 × 10^−7^ and *p* = 7.00 × 10^−7^). At the fifth and sixth peaks (tensors #4 and #5), no significant difference in the individual components among the clusters was observed (Figures 3C4,C5, *p* > 0.722).

Summarizing the above results, in the indirect phase of task achievement (i.e., the early movement and mid-flight phases), differences in the individual components among the clusters were found in tensors #1, #2, #3, and #6 (Figures 3B1–B3,B6). In the timing close to the direct phase of task achievement (the red shaded areas in Figures 3B1–B6), the differences disappeared in tensor #4 (Figure 3B4). In the transition phase from the indirect to direct phase of task achievement, there was no significant difference in the individual components among tensors in tensor #5 (Figure 3C5).

We thus speculated that the individual difference was evident in the indirect phase of task achievement and unnoticeable in the direct phase of task achievement. Similarly, common motion features could be obvious in the direct phase rather than the indirect phase. To quantify individually different recruitments of spatial and temporal modules, we calculated the variabilities of individual components within each tensor. If the within-tensor variability was small in some tensors, the associated spatial and temporal modules were commonly observable among the subjects. In contrast, large within-tensor variabilities in some tensors denoted individually different recruitments of the associated spatial and temporal modules.

Figure 3E supports the postulations that the individual difference was evident in the indirect phase and unnoticeable in the direct phase. The within-tensor variabilities of individual components were larger in the indirect phases (i.e., the early movement and mid-flight phases) than the direct phase to the task achievement (i.e., the hit phase). Because individual components denoted the amount of recruitment of the associated spatial and temporal modules, a larger within-tensor variability of the individual component indicated a larger individual difference. If all the subjects recruited the associated spatial and temporal modules in the same manner, the variability equaled 0. If all the subjects showed different recruitment patterns of the modules, the within-tensor variability of the individual component exhibited a large value. Figure 3E thus, indicates the existence of common and individually different motion components in the direct and indirect phases of task achievement, respectively.

To test these postulations from a different viewpoint, the current study segmented 500 time frames into five bins, each of which included 100 time frames, and performed the set of CP decomposition. After applying CP decomposition, we calculated the standard deviations of individual components within each tensor. Based on Figures 3C1–C6, a more obvious individual difference resulted in larger variability in the individual component [e.g., in tensor #3 (Figure 3C3)]. We then used the within-tensor variability as a measure of individual differences.

**Figure 4:**
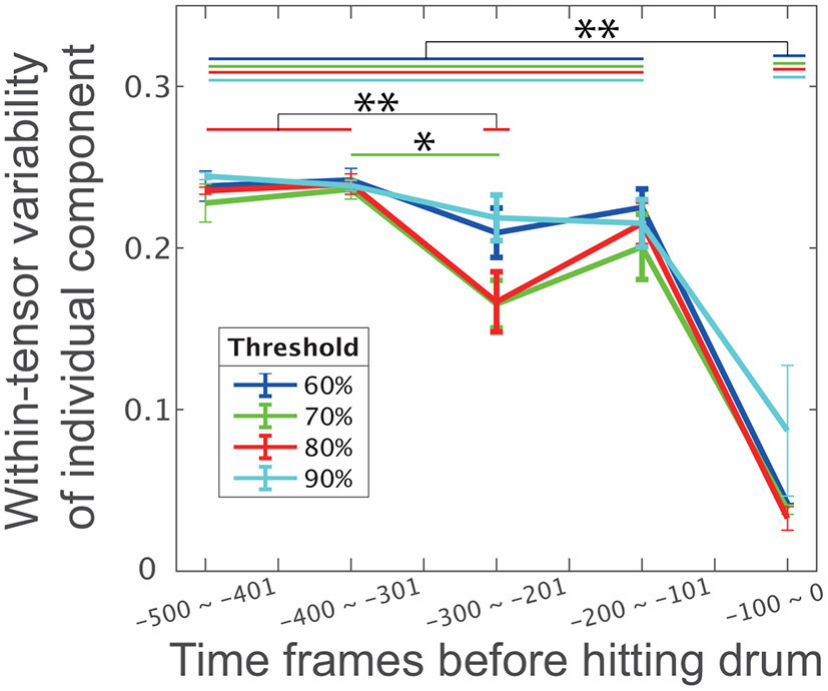
The influence of phase and threshold in CP decomposition on within-tensor variability of individual components. Error bars represent the within-tensor standard deviations of individual components; each of the bins includes 100 time frames. Blue, green, red, and cyan colors in this panel indicate 60, 70, 80, and 100% criteria, respectively. The single and double asterisks indicate significant differences via Tukey's comparison test at ^*^p < 0.05 and ^**^p < 0.01, respectively.

As expected, the within-tensor standard deviation was significantly smaller near the direct phase (i.e., the 5th bin) than in other phases (*p* = 2.46 × 10^−6^ [Tukey's comparison test], the black horizontal line with double asterisks between the 1st−4th bins and the 5th bin in Figure 4). The red line in Figure 4 demonstrates the error bar for within-tensor variability in each bin based on the criteria that we previously utilized (i.e., to explain more than 80% of the variance in the original data). We also found significant differences in the 3rd bin from the 1st and 2nd bins (*p* = 0.00762 and *p* = 0.00455 [Tukey's comparison test], denoted by horizontal red lines with double asterisks). These results indicated that the individual different motion features were obvious in the indirect phase (i.e., from the 1st to 4th bins) rather than the direct phase of task achievement (i.e., the 5th bin). Similarly, common motion features were more evident in the direct phase rather than the indirect phase.

We then assessed the influence of the number of tensors on our findings to further validate our assumption: individual differences are evident in the indirect phase rather than the direct phase of task achievement, and common features are obvious in the direct phase rather than the indirect phase of task achievement. To determine the number of tensors in the CP decomposition, this study used the criteria of 80% to explain the variance in the original data. Although six tensors were extracted with this value, the number of tensors could change depending on the criteria. To examine whether our current finding persist irrespective of the criteria, we performed CP decompositions for four criteria: 60, 70, 80, or 90%.

Along with our expectation, our results were invariant independent of the criteria (Figure 4). The within-tensor variability in the individual components was significantly smaller at the 5th bin than the 1st−4th bins (*p* < 0.0022 [Tukey's comparison test], the black horizontal line with double asterisks between the 1st and 4th bins and the 5th bin in Figure 4). The blue, green, red, and cyan lines in Figure 4 denote the within-tensor variability in each bin based on 60, 70, 80, and 90% criteria, respectively. We also found a significant difference between the 2nd and 3rd bins at the 70% criteria (*p* = 0.0422 [Tukey's comparison test], denoted by the horizontal green line with a single asterisk). We thus concluded that individual differences were evident in the indirect phases and that common features were obvious in the direct phase of task achievement.

**Figure 5:**
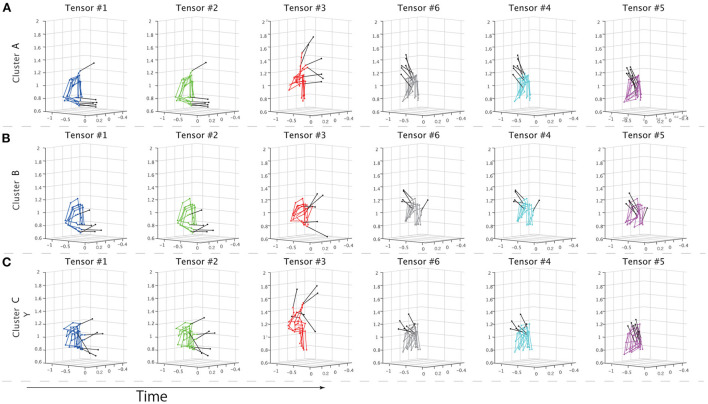
Properties of extracted clusters. **(A–C)** The non-standardized motion data of all the subjects in each cluster. The solid black lines indicate drumsticks. Each panel shows the movements at the times of the peaks in the temporal modules. The colors denote the tensor numbers, as in Figure 3. From left to right, the tensors are sorted based on the peak timings. The left panel shows the drum-hitting motions close to the early movement phase, and the right panel shows the motions close to when the drums were hit. **(A–C)** Show the drum-hitting motions in clusters A, B, and C, respectively.

To further evaluate the speculations from different perspectives, non-standardized motions at the times of the peaks for each temporal module were visualized in Figure 5. We sorted the motions based on the peak timings; the left panels show the earlier peaks, and the right panels show the later peaks. Supplementary Movie 1 shows the time-varying motions with highlights in the same manner as Figure 5. Figures 5A–C demonstrates the non-standardized motions in clusters A-C. We found obvious individually different motion patterns in the indirect phase (e.g., blue-, green-, and red-colored). At the time shown in the left-most panels, the subjects classified in cluster C had already started stick-raising motions. The subjects in clusters B and A had not yet started the motions. The tendency was consistent in the panels colored blue and green. These across-group differences in motion phases were likely reflected by the differences in individual components (Figures 3C1,C2). In the middle part of the indirect phase (red-colored), the subjects in cluster B started to raise their drumsticks, but the other subjects had already finished the raising motion. Near the timings close to the direct phase (gray-, cyan-, and magenta-colored), few obvious individual differences were noted.

Although we focused on drum-hitting motions with maximum effort, whether the same tendencies were consistent in minimum-effort motions remained unclear. In contrast to the maximum-effort motions, the minimum-effort motions had few individual differences: the number of clusters was one based on gap value criteria (Supplementary Figure 2B). In the minimum-effort motions, all the subjects showed slight motions to raise the drumstick and no other movements. All the minimum-effort motions could be classified as the direct phase of task achievement. Along with the results mentioned previously, few individual differences in the minimum-effort motions indicated that common motion features were evident in the direct phase of task achievement.

## Discussion

The current study investigated the relationships among two features inherent in skilled movements: (1) common and individually different features of motions and (2) direct and indirect phases of task achievement. We utilized both drum-hitting motions performed by professionals (Figure 2A) and tensor decomposition (Figure 1). With drum-hitting motions, we were able to investigate how common and individually different features appeared in the direct phase (i.e., hit phase) and indirect phase (i.e., the early movement and mid-flight phases; Figures 2B, 3–5). Tensor decomposition enabled us to assess three factors: spatial modules, temporal modules, and how the recruitment patterns of the modules are common or individually different. We verified that common spatiotemporal modules are evident in the direct phase and that individually different spatiotemporal modules are evident in the indirect phase of task achievement (Figures 3–5).

A previous study investigated individual differences in drum-hitting motions (Dahl, 2004). The study showed a clear correlation between the preparatory height of a stick in mid-flight and the velocity of a stick in hitting a drum. Furthermore, the correlation values were similar across all four subjects. These uniform correlations indicated that the preparatory stick height affects the stick hitting velocity. In our study, all the subjects showed similar movements when hitting a drum (Figures 3D,E, 5, Supplementary Movie 1). If the correlation between stick preparatory height and hitting velocity is consistent between the previous and current studies, we would expect the same preparatory stick heights in all the subjects. In contrast to this hypothesis, all the subjects seemed to show different preparatory stick heights (Figure 5, Supplementary Movie 1). This dissimilarity between the previous and current studies can be attributed to the difference in the early movement phase. In a previous study, the subjects performed drum-hitting motions in response to periodic sounds. Thus, all the subjects had almost the same early movement phase and termination times associated with hitting the drums. In contrast, we did not control this timing such that we could assess individual differences with few restrictions. In our study, some subjects raised the stick in an early phase (e.g., the subjects included in clusters A and C in Figures 5A,C, see Supplementary Movie 1), and other subjects did not raise the stick until the mid-flight phase (e.g., the subjects included in cluster B in Figure 5B, see Supplementary Movie 1). In other words, the preparatory stick height is more difficult to define in our study than in the previous study.

We have already demonstrated the effectiveness of tensor decomposition, or CP decomposition, in assessing task-dependent modulations (Takiyama et al., 2020b; Hashimoto et al., 2021) and individual differences (in this study) inherent in spatial and temporal modules. Tensor decomposition enables us to assess diverse types of modulations or differences according to how the tensor data are generated. Because we measured drum-hitting motions with various levels of effort in our study (see Section Materials and methods for details), we were able to assess effort-dependent modulations of spatiotemporal modules as in our earlier study (Hashimoto et al., 2021). In this case, we need to generate tensor data with dimensions for the body part, time, and effort factors; the matrix size is *I* × *J* × *L*, where *L* is the number of tasks performed with different levels of effort. The third dimension becomes effort-dependent components rather than individual components. Other applications of this method include assessing the difference between amateur and professional drummers or the difference between musicians with and without neurological disease. Learning- or adaptation-dependent modulations of spatiotemporal modules can also be discussed using tensor decomposition. Because several computational models have been proposed for motor adaptation with arm-reaching movements (Thoroughman and Shadmehr, 2000; Takiyama et al., 2015; Takiyama and Sakai, 2016; Ishii et al., 2018), identifying the relationships between adaptation models and adaptation-dependent modulations extracted by tensor decomposition may be useful in future work.

Although we focused on CP decomposition, various versions of tensor decomposition exist. For example, the assumption of smooth temporal variation can be introduced in temporal modules (Takiyama et al., 2009; Takiyama and Okada, 2011; Naruse et al., 2013). Another common method is Tucker decomposition (Kolda and Bader, 2009). In Tucker decomposition, the number of spatial modules, temporal modules, and the third component (e.g., individual component) can differ from each other. In contrast, in CP decomposition, these numbers are the same. Due to its flexibility and complexity, Tucker decomposition allows us to, for example, estimate a spatial module common to two temporal modules and three individual factors. For complex analyses such as neural activity analyses, Tucker decomposition can be used to extract low-dimensional structures inherent in data (Onken et al., 2016). Additionally, due to the flexibility of Tucker decomposition, we need to determine three free parameters: the number of spatial modules, temporal modules, and individual components. Because several combinations of the parameters result in the same variance explained, determining the number of parameters is still an ongoing problem (Delis et al., 2014, 2018). Due to the simplicity of CP decomposition, we need to determine only one parameter: the number of combinations of a spatial module, a temporal module, and an individual component. Although Tucker decomposition can be powerful in a more complex situation, we utilized CP decomposition due to its simplicity.

## Data availability statement

The raw data supporting the conclusions of this article will be made available by the authors, without undue reservation.

## Ethics statement

The studies involving human participants were reviewed and approved by Ethical Committee of the Graduate School of Arts and Sciences of the University of Tokyo. The patients/participants provided their written informed consent to participate in this study.

## Author contributions

SF performed all data measurements. MH and SF preprocessed the data and reviewed the paper. KT analyzed the data. KT and SF wrote the paper. All authors contributed to the article and approved the submitted version.

## Funding

We acknowledge support from the Japanese Society for Promoting Science [Grant-in-Aid for Scientific Research (B) (20H04089) to KT and Grant-in-Aid for Scientific Research (B) (20H04092) to SF].

## Conflict of interest

The authors declare that the research was conducted in the absence of any commercial or financial relationships that could be construed as a potential conflict of interest.

## Publisher's note

All claims expressed in this article are solely those of the authors and do not necessarily represent those of their affiliated organizations, or those of the publisher, the editors and the reviewers. Any product that may be evaluated in this article, or claim that may be made by its manufacturer, is not guaranteed or endorsed by the publisher.

## Supplementary material

Supplementary Movie 1 A movie demonstrating the classification of drum-hitting motions into three clusters. The phases around the peak timings for each temporal module are slowed down to highlight the timings. Because the peak timings of tensor #1 and #2 were close to each other and the peak timing of tensor #1 was within the 1st time frame, long highlights for both tensors did not provide effective information. We thus set the highlight to be shorter for tensor #1.
