# Supplementary material for: Transition between individually different and common features in skilled drumming movements
Source: Front Sports Act Living. 2022 Jul 26;4:923180. doi: 10.3389/fspor.2022.923180 (PMC9361045; doi:10.3389/fspor.2022.923180)
Supplement: Supplementary file 2 [file Data_Sheet_1.pdf]

Supplementary Information

Transition between individually different and common features in skilled drumming movements

Ken Takiyama[1*], Masaya Hirashima[2], Shinya Fujii[3]
1: Tokyo University of Agriculture and Technology, Department of Engineering, Electrical and Electronic Engineering, Koganei, 2-24-16, Tokyo, Japan
2: Center for Information and Neural Networks (CiNet), National Institute of Information and Communications Technology, and Osaka University, Suita, Osaka 565-0871, Japan
3: Keio University, Faculty of Environment and Information Studies, Fujisawa, Kanagawa, 252-0882, Japan

* Correspondence should be addressed to K.T. (t.j.ken.takiyama@gmail.com).

Importance and invariance of our results independent of standardization

To apply Candecomp/Parafac (CP) decomposition appropriately, we standardized motion data. Let $X_{i,j,k}$ be motion data including the information on the ith marker position (e.g., in the case of the main text, i = 1 indicates the forward-backward position of the stick marker, and i = 2 indicates the up-down position of the stick marker), jth time frame, and kth subject. For standardization, we calculated the mean $m_{i,k} = \frac{1}{J}\sum_{j=1}^{J} X_{i,j,k}$ and the standard deviation $s_{i,k} = \sqrt{\frac{1}{J}\sum_{j=1}^{J}\left(X_{i,j,k} - m_{i,k}\right)^2}$ across time frames in each marker position and subject. Next, we standardized motion data as

$$\tilde{X}_{i,j,k} = \frac{X_{i,j,k} - m_{i,k}}{s_{i,k}} \dots (1)$$

such that the mean and standard deviation of $\tilde{X}_{i,j,k}$ across time frames are 0 and 1, respectively. Finally, we applied CP decomposition to the standardized motion data. Of note, without standardization, CP decomposition prioritizes components whose mean and standard deviation are large and neglects components whose mean and standard deviation are small. To fairly discuss all motion features, the standardization process is indispensable.

Under the standardization process, the standardized motion data are decomposed as

$$\tilde{X}_{i,j,k} \simeq \sum_{\mathrm{r}=1}^{\mathrm{R}} \lambda_r\, s_{i,r} t_{j,r} u_{k,r}, \dots (2)$$

i.e., $\tilde{X}_{i,j,k}$ is decomposed into R combinations of a weight coefficient, a spatial module, a temporal module, and an individual component. From a different viewpoint, Eq. (2) means that $\tilde{X}_{i,j,k}$ can be reconstructed by the R combinations. By inserting Eq. (1) into Eq. (2), the nonstandardized motion data can be written as

$$X_{i,j,k} \simeq m_{i,k} + s_{i,k} \sum_{r=1}^{R} \lambda_r \, s_{i,r} t_{j,r} u_{k,r},$$

indicating that the original motion data can be reconstructed by using extracted spatial modules, temporal modules, and individual components. Thus, the standardization process does not significantly affect our results.

Invariance of our results in the analysis of joint angles

Because we analyzed the positions of representative markers in our main manuscript, it remained unclear whether the results were invariant in the analysis of joint angles. We thus calculated joint angles and applied CP decomposition to the joint angle data (Fig. S1). The joint angle data included two stick movements (i.e., parallel or orthogonal to the plane including the proximal finger joint and wrist), three wrist movements (i.e., extension-flexion, abduction-adduction, and pronation-supination), one elbow movement (i.e., extension-flexion), three shoulder movements (i.e., extension-flexion, abduction-adduction, and medial-lateral rotation), and one trunk movement (i.e., raised angle of the right shoulder). Of note, the joint angle analysis provided a smaller number of tensors than the analysis of marker positions. Under the same criteria as that defined in the main text (i.e., 80% variance of the original motion data explained), the extracted number of tensors was 2. Because it was impossible to perform comparisons in the case of 80% criteria for the joint angle analysis, we set the criteria to be 95% and extracted 6 tensors. As shown in Fig. S1D, the index of individual difference showed the same tendency, specifically, a smaller individual difference in the direct phase and a larger individual difference in the indirect phase of task achievement. In the analysis of joint angles, there were few individual differences at the earliest part of the movements (the leftmost circle in Fig. S1D).

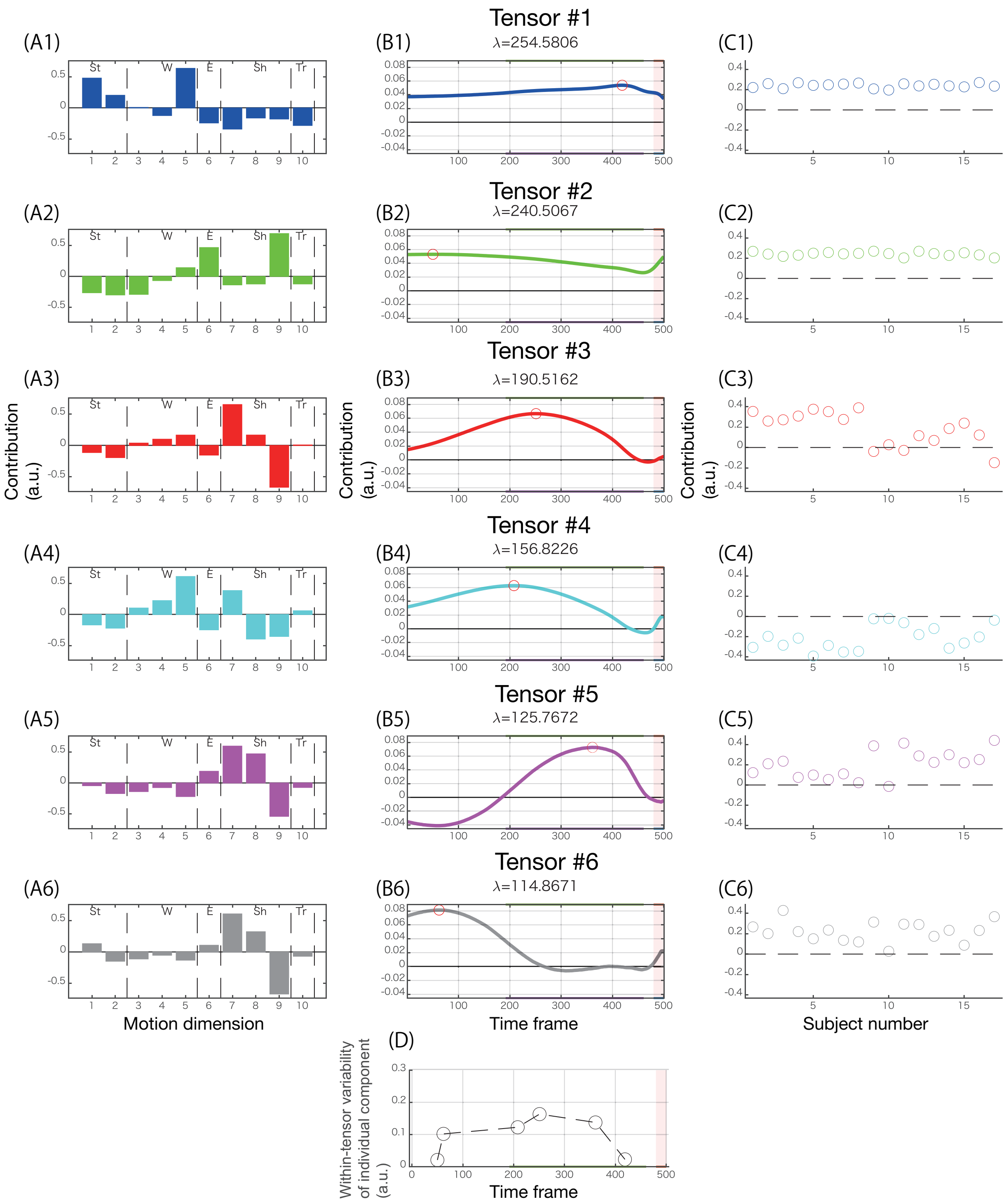

Figure S1: **Tensors extracted via CP decomposition in the joint angle analysis**. The bar graphs indicate spatial modules (panels A1-A6), the line plots denote temporal modules (panels B1-B6), and the scatter plots show individual components (panels C1-C6). λ is a scaling factor indicating how each tensor contributes to the reconstruction of the original data.

All the spatial modules, temporal modules, and individual components were normalized such that each norm equaled 1. (Left panels): In the spatial modules, S represents sticks, F represents fingers, W represents wrists, E represents elbows, Sh represents shoulders, Ut represents the upper part of the trunk, and Lt represents the lower part of the trunk. In each segmentation, three bins indicate the marker positions in the x- (forward-backward direction), y- (upward-downward direction), and z-coordinates (rightward-leftward direction) from left to right. (Middle panels): In the temporal modules, the 500th time frame corresponds to the time at which the drum was hit. Each black circle indicates the time of the peak of each temporal module. Nonstandardized motions at these peak timings are shown in Fig. 4. The red shaded areas in the same panels demonstrate the phases when the magnitude of the stick up-down velocity was significantly different from 0 (see Fig. 2B). (C1-C6): The individual components indicate how spatiotemporal modules are recruited by each subject. Of note, the subject number was sorted based on the cluster number in a *post hoc* manner to increase visibility. Each cluster is separated by horizontal dotted black lines. The single and double asterisks above the horizontal solid black lines indicate significant differences via Tukey's comparison test at $p < 0.05$ and $p < 0.01$, respectively. (D): Within-tensor variability of each tensor based on the peak timing of each temporal module.

Gap values for hierarchical clustering

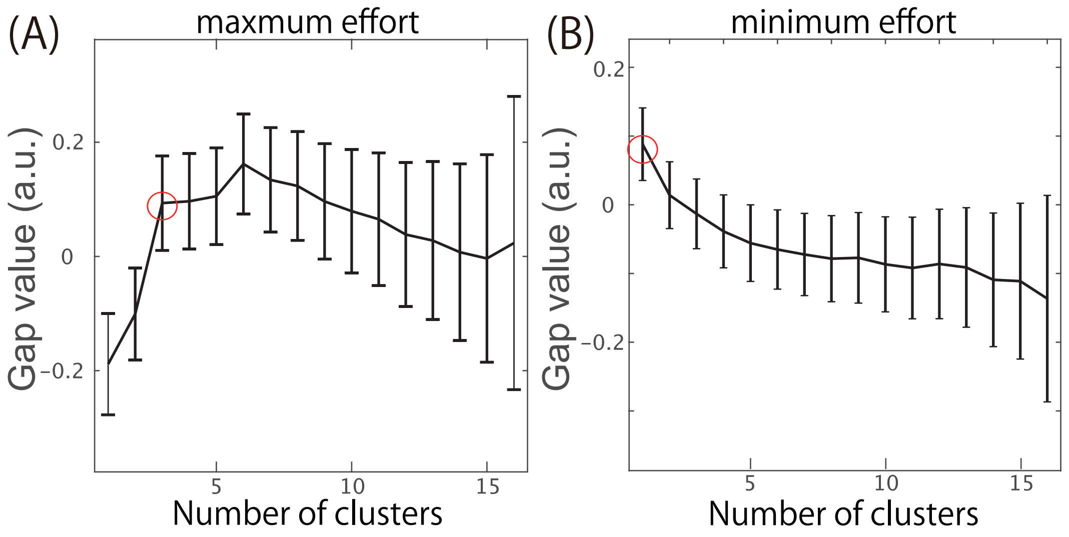

Figure S2: **Gap values for hierarchical clustering**. (A): The gap values for hierarchical clustering of individual components when subjects hit a snare drum with their maximum effort. (B): The gap values for hierarchical clustering of individual components when subjects hit a snare drum with their minimum effort.

Task performance

Because subjects were instructed to gradually increase motion effort in each set of trials, a measure of task performance was sound volume in each trial. If they performed the task in an ideal manner, they hit a snare drum with a larger sound volume in later trials. Because we did not explicitly determine the number of trials in each set (please see the Materials and Methods section in our main text), the subjects determined the number of trials by themselves. To examine the relationship between the trial number and sound volume, the number of trials was different in each set and in each subject. Additionally, sound volume differed, possibly according to height, muscle strength, and arm length. We thus normalized the trial number and sound volume in each set such that the maximum values of trial number and sound volume were 1 in each set and in each subject.

The signal of the microphone was amplified (M1204, ATL), mixed (LS9-16, YAHAMA), converted from analog to digital at a frequency of 48,000 Hz (Fire Wire 410, M-Audio), and recorded on a personal computer (iBook G4, M9846J/A, Apple) using ProTools software (M-Powered v7.3, Digidesign).

Fig. S3 demonstrates a clear increase in sound volume depending on the trial number. In other words, subjects successfully increased their motion effort gradually in each trial. Of note, we analyzed 10 of 17 subjects because the recording accuracy was not sufficient to extract the exact hit timings in several trials.

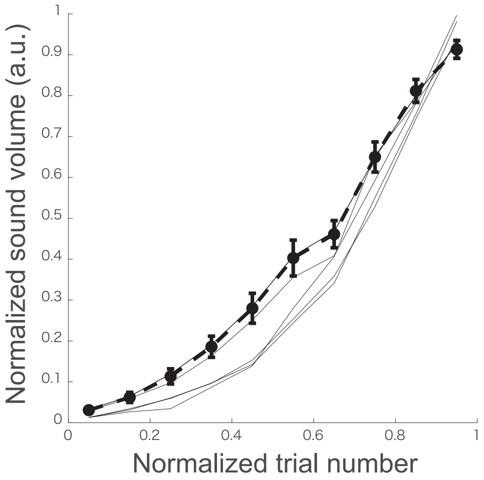

Figure S3: **Sound volume in each trial**. The horizontal line indicates the trial number divided by its maximum value. The vertical line indicates the sound volume divided by its maximum value. Because normalized trial numbers differ in each set and subject, we further divided normalized trial numbers into ten intervals (0-0.1, 0.1-0.2, …, 0.9-1). We then calculated the mean and standard error of the mean within each interval in each set and subject. Thin black lines indicate the mean sound volume in each subject (N = 10). The black dotted lines and error bars denote the mean and standard error of the mean sound volume across all sets and subjects.

Attributes of subjects

Table S1: Attributes of subjects.

Subject Number	Age	Years of experience	Average practice time per week (hours)	Sex	Cluster
1	58	43	12.1	M	A
2	43	30	21.9	M	A
3	44	29	17.7	M	A
4	32	16	10.5	M	A
5	43	28	17.5	M	A
6	42	28	17.6	M	B
7	37	20	11.7	M	B
8	47	39	50.8	M	B
9	42	31	31.4	M	B
10	26	11	12.9	F	B
11	40	25	13.8	M	B
12	43	29	23.8	M	C
13	45	29	50.7	M	C
14	34	18	16.3	M	C
15	43	37	19.7	F	C
16	23	8	16.1	M	C
17	40	24	9	M	C

There was no significant difference in the attributes among the clusters ($p > 0.8572$ [corrected pairwise comparisons]).
